# A upconversion luminescene biosensor based on dual-signal amplification for the detection of short DNA species of c-erbB-2 oncogene

**DOI:** 10.1038/srep24813

**Published:** 2016-04-21

**Authors:** Jianming Lan, Yingxin Liu, Li Li, Fadi Wen, Fang Wu, Zhizhong Han, Weiming Sun, Chunyan Li, Jinghua Chen

**Affiliations:** 1 Department of Basic Chemistry, The School of Pharmacy, Fujian Medical University, Fuzhou, Fujian 350108, P. R. China; 2 Department of Pharmaceutical Analysis, The School of Pharmacy, Fujian Medical University, Fuzhou, Fujian 350108, P. R. China

## Abstract

High-sensitivity detection of trace amounts of c-erbB-2 oncogene was reported to be equal to or surpass the ability of CA 15-3 for early diagnosis and/or follow-up recurrent screening of breast cancer. Therefore, in the current study, by using upconversion nanoparticles (UCNPs), rare earth-doped NaYF_4_:Yb^3+^/Er^3+^ as the luminescent labels, a upconversion luminescent (UCL) biosensor based on dual-signal amplification of exonuclease III (ExoIII)-assisted target cycles and long-range self-assembly DNA concatamers was developed for the detection of c-erbB-2 oncogene. The proposed biosensor exhibited ultrasensitive detection with limit as low as 40 aM, which may express the potential of being used in trace analysis of c-erbB-2 oncogene and early diagnosis of breast cancer.

In many tumor diseases, breast cancer is one of the most multiple malignant tumors in women. Recent studies have indicated that early detection, diagnosis and treatment of breast cancer are the key problems needed to prevent and reduce mortality rate. In the process of tumor initiation and proliferation, tumor cells synthesize and release a substance called the tumor marker, which results from host cells responding to the tumor. Recently, studies have found that it’s very difficult to detect anomalies in the early stages of tumor by usual methods, but the trace amounts of tumor marker in the blood would present proliferation in certain extent. Therefore, the tumor marker test is the most effective way for diagnosis cancer in the early asymptomatic stage^1^. Presently, the medical researchers have found a vast variety of markers^2^ associated with breast cancer, such as proteins, genes and growth factors, etc. Among them, soluble fragments of the c-erbB-2 oncogene, located on chromosome 17, q21, can be released from the cell surface and become detectable in the body fluid (serum or saliva) of breast cancer patients^3^. Present studies suggest that the overexpression of c-erbB-2 oncogene usually indicates cancer cell proliferation^4^,^5^. Thus, as a new breast cancer-associated biomarker, c-erbB-2 oncogene is expected to replace CA 15–3, becomes the gold standard for early diagnosis and/or follow-up recurrent screening of breast cancer^6^. But even so, in the early diagnosis of breast cancer, the effect of direct detection is poor due to generally low concentration of c-erbB-2 oncogene in the actual samples. Therefore, how to improve the detection sensitivity becomes very important.

Recently, the researchers pay more and more attention to the application of signal amplification detection technology^7^. Since the mid 1980s, Polymerase chain reaction technology (PCR) was being widely used in clinical applications because of its significant signal amplification. But this technology includes the potential defects, such as easy to cause false positive, vulnerable to pollution and so on^8^. Rolling circle amplification (RCA)^9^ and bio-bar code assay (BCA)^10^ were mainly new technique emerged in recent years, which were of great significance for improving the detection sensitivity, but they had some shortages, such as complicated operation time-consuming and expensive cost.

To improve the above issue, many strategies based on the enzyme-catalyzed target-recycling signal amplification have been developed and applied for realizing the ultrasensitive DNA detection. For example, exonuclease III (ExoIII) has been employed as enzyme-catalyzed target-recycling signal amplification for ultrasensitive DNA detection because of its excellent characteristics^11^. Some reported ExoIII-assisted signal amplification strategies demonstrated that the signaling probes could be selectively digested by ExoIII with the target DNA cycling, and finally achieved signal amplification^12^. These ExoIII-based methods are sensitive and selective for the detection of target DNA in appropriate conditions. At the same time, in order to further improve the sensitivity and selectivity of DNA biosensor, DNA concatamer, one of linear polymeric structures formed by self-association of short DNA fragments through specific interactions, has caught strong attention in DNA diagnostics^13^. Moreover, their branched 2D or 3D analogs have already been applied to amplify the signal and enhance trapping of the query nucleic acid in hybridization analysis^14^. Recently, the combination of enzyme-assisted target cycle and DNA concatamer were also reported for the signal amplification of DNA sensors^15^. During the process, high sensitivity was achieved by using labeling materials (e.g. organic dyes, metal complexes, quantum dots or fluorescent proteins) in the DNA concatamers^16^,^17^,^18^. Typically, the signal increases with an increase in amount of labels to some extent. However, these conventional photoluminescence (PL) imaging agents have several insuperable limitations, such as low signal-to-background ratio (SBR) caused by auto-fluorescence of biological tissues when excited by short wavelengths, low penetration depth of ultraviolet (UV) and visible excitation and/or emission light in biological tissues, and potential DNA damage and cell death due to long-term exposure to short wavelength, particularly under the UV excitation^19^. Therefore, it is necessary to find a new labeling mediator to overcome these shortcomings.

To date, because of the excellent physicochemical properties such as superior chemical stability, sharp-band emissions, large anti-Stokes shifts, long lifetimes, low toxicity and high resistance to photobleaching^20^,^21^,^22^, NaYF_4_:Yb^3+^/Er^3+^ upconversion nanoparticles (UCNPs) have been further developed as a new generation of bioprobe and applied to the field of biomedicine. Moreover, the use of near-infrared (NIR, 980 nm) CW light excitation can effectively avoid photodamage to living organisms and the autofluorescence of some biological samples^23^,^24^,^25^. However, it will be great challenge to obtain functionalized UCNPs with good hydrophilic and biocompatible, as they are usually coated with inert hydrophobic ligands after synthesis. To satisfy these requirements, many efforts have been developed to the surface modification and bioconjugation of UCNPs with different hydrophilic group, such as Ligand exchange^26^, ligand oxidation^27^, surface silanization^28^, one-step solvothermal synthesis^29^ and silica coating^30^. Recently, Lu group reported a facile approach to prepare DNA-functionalized NaYF_4:_Yb^3+^/Er^3+^ UCNPs based on ligand exchange at the liquid–liquid interface^31^, which led to the formation of bioconjugates that retained the characteristic of both DNA and UCNPs. Consequently, it can be sure that DNA-UCNPs should be regarded as a promising class of luminescent probes with high sensitivity and low background fluorescence. Further, in order to improve the sensitivity of the probe, it is necessary to employ the luminescent signal amplification technology.

In this paper, it is hopeful to find a simple, noninvasive and highly sensitive detection for the c-erbB-2 oncogene. Combining the above methods with previous reports, we would design a new UCL biosensor for the detection of c-erbB-2 oncogene based on dual-signal amplification of ExoIII-assisted target cycles and long-range self-assembly DNA concatamers combined with UCNPs.

## Results and Discussions

### Characterization of NaYF_4_:Yb^3+^/Er^3+^ UCNPs

NaYF_4_:Yb^3+^/Er^3+^, one of the most efficient UCNPs, was selected as the energy donor for this biosensor. The precise control of high-quality nanocrystals is especially important for the sensitivity of biosensor. Therefore, the size, crystal phase purity, structural constituent, morphology and luminescent property of the resulted NaYF_4_:Yb^3+^/Er^3+^ UCNPs were characterized in detail by the corresponding methods.

**Figure 1:**
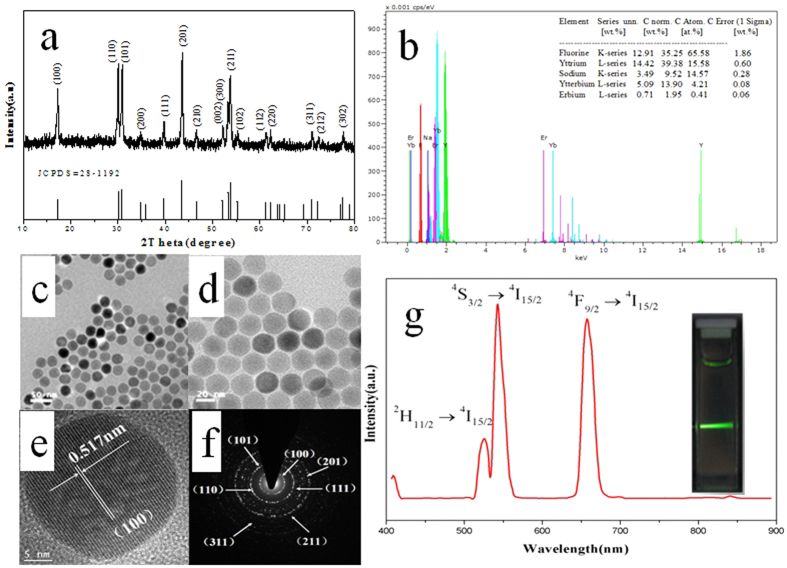
Characteristics of as-synthesized NaYF_4_:Yb^3+^/Er^3+^ UCNPs. (**a**) Experimental powder X-ray diffraction (XRD) pattern (top curve) and the calculated line pattern for β-NaYF_4_ (bottom curve); (**b**) X-ray energy dispersive spectrum (EDS); (**c**,**d**) TEM images; (**e**) HRTEM images; (**f**) SAED pattern; (**g**) The UCL spectrum. Inset: Photograph of the UCNPs dissolved in cyclohexane (1%, w/V) and excited with a 980 nm laser.

The typical XRD pattern (Fig. 1a) corresponds almost exactly with the standard pattern of the hexagonal phase (β) NaYF_4_ (JCPDS No. 028-1192) without other peaks of cubic phase (α), suggesting that the nanoparticles belong to pure hexagonal phase and have fine crystallinity. The atomic composition ratios of the lanthanides in NaYF_4_:Yb^3+^/Er^3+^ UCNPs were determined by EDS. As shown in Fig. 1b, the measured atomic ratios of the lanthanide elements (Y: Yb: Er = 15.58: 4.21: 0.41) are very close to the calculated values (Y: Yb: Er = 0.78: 0.2: 0.02), indicating that we can control the constituents during the nanocrystal growth effectively. As shown in Fig. 1c,d, the UCNPs appear approximately spherical shape and good dispersibility. And it can be found that UCNPs are of fine single crystalline nature based on Fig. 1e. The lattice fringes can be clearly distinguished from the HRTEM images. And the value 0.517 nm between the lattice fringes is belong to the d spacing for the (100) lattice plane. The selected-area electron diffraction (SAED) pattern (Fig. 1f) shows that spotty polycrystalline diffraction rings can be obtained corresponding to the (100), (101), (110), (111), (201), (211), and (311) planes of the β-NaYF_4_:Yb^3+^/Er^3+^ lattice. The upconversion specta of 1 wt% solution of UCNPs in cyclohexane under 980 nm excition are shown in Fig. 1g. There are three emission centers at about 521 nm, 541 nm and 656 nm, which are attributed to the ^2^H_11/2_ → ^4^I_15/2_, ^4^S_3/2_ → ^4^I_15/2_ and ^4^F_9/2_ → ^4^I_15/2_ transitions of Er^3+^ ions, respectively. The inset in Fig. 1g demonstrates that the green light can be easily observed by the naked eye or other imaging systems, which suggests that the NaYF_4_:Yb^3+^/Er^3+^ UCNPs are potential candidate materials for biomarker. The emission peak at 656 nm is chosen as the testing signal in our detection, which can effectively avoid the autofluorescence of biological samples.

### DNA modification of NaYF_4_:Yb^3+^/Er^3+^ UCNPs

In order to enhance the dispersibility in water, NaYF_4_:Yb^3+^/Er^3+^ UCNPs were modified with DNA via the strategy of the single-stranded DNA self-assembled functionally. As shown in Supplementary Fig. S1, the DNA can be confirmed by the observation of typical UV absorbance at 260 nm. After DNA modification, an obvious absorption peak at the same location was observed for AP2-UCNPs, but the intensity of UV absorption peak nearly reduced by half compared to DNA itself. And the UCNPs in the chloroform had no absorption peak at 260 nm. In addition, the AP2-UCNPs aqueous solution was also verified by the zeta potential. The measurement showed that the zeta potential of UCNPs was −31.80 mv after AP2 modified. Therefore, we can conclude that the OA molecules on the surface of 25 nm β-NaYF_4_:Yb^3+^/Er^3+^ UCNPs can be successfully replaced by DNA.

### The test mechanism of UCL biosensor for c-erbB-2 oncogene

With NaYF_4_:Yb^3+^/Er^3+^ UCNPs as probe, a highly sensitive and specific UCL biosensor based on dual signal amplification of ExoIII-assisted target cycles and DNA concatamers was developed for the detection of c-erbB-2 oncogene (T1). Firstly, the hairpin capture probes (CP) were fixed on the microplates. When T1 was present, the first signal amplification was achieved through the process of hybridization, degradation, and rehybridization with the aid of the ExoIII. Secondly, the addition of two auxiliary probes AP1 and AP2 could trigger the formation of super sandwich DNA concatamers by 1ong-range self-assembly. Then the above prepared structure could generate a strong UCL signal through dual signal amplification when being excited by 980 nm because of the AP2 modificated with UCNPs. The ultra high sensitivity detection of T1 can be realized by detecting the intensity change of UCL signal in the presence or absent of T1.

**Figure 2:**
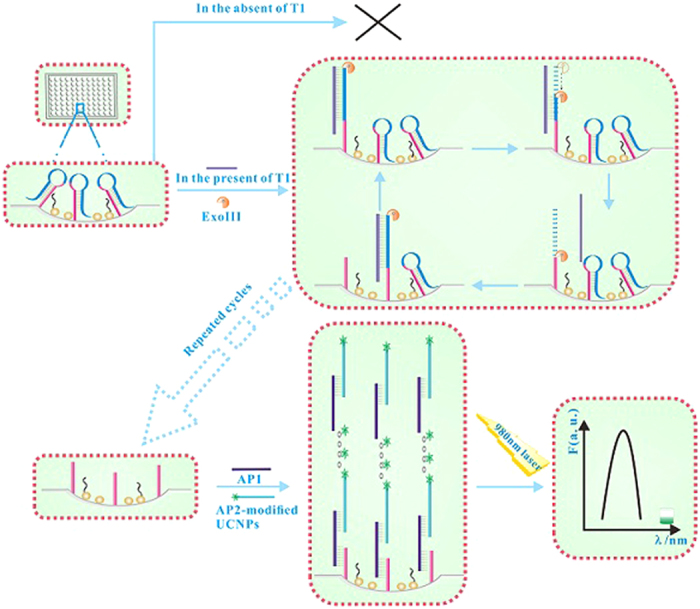
Experimental schematic of the designed biosensor.

The process was sketched in Fig. 2. The ExoIII was chosen as the tool enzyme for signal amplification of ExoIII-assisted target cycle. The mononucleotide of double-stranded DNA can be effectively degraded with 3′ → 5′ directionality due to the action on 3′ blunt ends of double-stranded DNA by ExoIII. Otherwise, it is less active on single-stranded DNA or 3′ protruding ends of double-stranded DNA. Firstly, the hairpin CP is assembled on the surface of 96 pore plates to form stem due to 3′ protruding ends. When the target T1 arises, CP can be hybridized with T1 to form double-stranded DNA structure with 3′ blunt ends. Then, CP can be gradually degraded from 3′ blunt ends of double-stranded DNA by ExoIII until double-stranded structures (hybridized with T1) are completely degraded. Finally, the fragment residues of free DNA are released on the surface of 96 pore plates. Meanwhile, another CP can be hybridized again by the entirely released T1. Consequently, after repeating continuous cycles of hybridization-digestion-rehybridization, many strands of CP can be degraded by one T1 DNA strand theoretically. When the above cycles completed, the CP should be truncated from hairpin structure to flexible short-chain one. Further adding AP1 and AP2, T1 can implement a process of concatamer hybridization with AP1 and AP2. Finally, the long-range self-assembled DNA nanostructures are fixed on the surface of the microplates. Because NaYF_4_:Yb^3+^/Er^3+^ UCNPs are modified on AP2, the long-range self-assembled DNA nanostructures should generate a remarkable amplified UCL signal excited by 980 nm. Whereas, the DNA nanostructures self-assembled by AP1 and AP2 can’t be coupled on the surface of the microplates due to the closure of red sequences of CP without the hybridization of T1. Thus only weak fluorescence signal can be detected on account of a small amount of nonspecific adsorption. Finally, the ultrasensitive detection of T1 can be realized by comparing the intensity change of UCL signal according to the different amounts of T1.

### Standard curve for UCL measurement of biosensor

**Figure 3:**
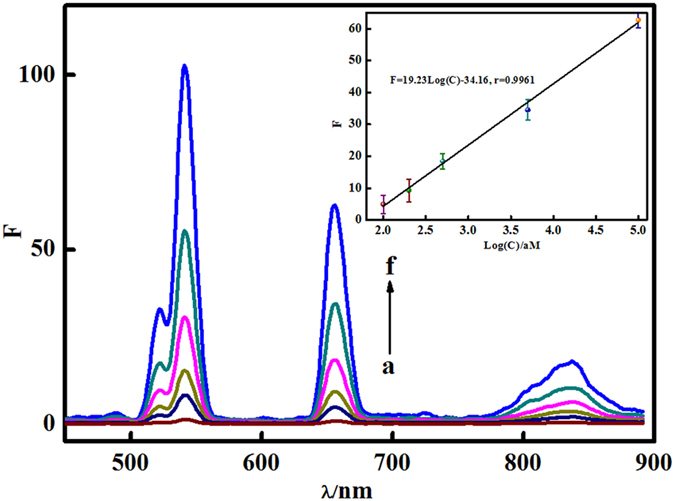
UCL detection for c-erbB-2 oncogene with different concentrations: 0 aM, 100 aM, 200 aM, 500 aM, 5fM, 100 fM (From a to f). (Inset: The linear fit plot of UCL intensity and the logarithm of concentration of T1.)

As shown in Fig. 3, under the optimal experimental conditions (as shown in Supplementary), the UCL intensity (F) gradually increases with the concentrations of T1 from 100 aM to 100 fM. The fitted data show that F is be linear with the logarithm of concentration of T1, the correlation equation can be expressed as F = 19.23log(C)-34.16 (r = 0.9961). According to calculation, the limit of detection (LOD) based on 3ơ method as low as 40 aM can be achieved. Then, 100 aM c-erbB-2 was chosen to investigate the precision of the proposed method by repeating each group test for 5 times. Conclusively, the as-obtained relative standard deviation (RSD) tested in the uniform plate and different plates are 2.82% and 3.56%, respectively, which shows that the developed biosensor exhibited good reproducibility and acceptable stability. Recently, many researches have focused on the different signal amplification techniques to detect DNA, and the reported corresponding LODs are 10 nM^32^, 10 pM^33^, 0.167 pM^34^, 0.1 pM^35^, and 30 fM^36^ respectively (More details shown as in Supplementary Table S1). Compared with the above research, our proposed UCL biosensor based on dual signal amplification of ExoIII-assisted target cycles and long-range self-assembly DNA concatamers shows high sensitivity and stability. Therefore, this UCL biosensor would provide a simple way for the ultra low detection of c-erbB-2 oncogene in clinical sample.

### Specificity and stability of biosensor

**Figure 4:**
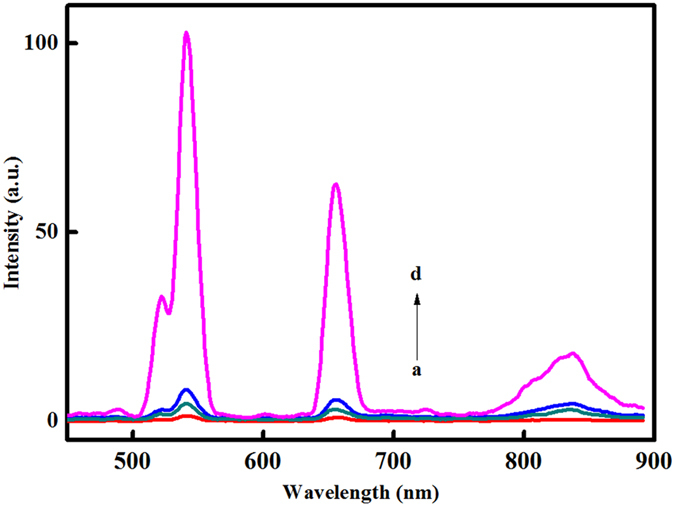
Specificity of the biosensor for the blank and three various RNA sequences (a. blank; b. NC; c. 1MT, d. T1).

In order to examine the specificity of the proposed UCL biosensor, we performed a comparison study between mismatch targets and perfect complementary target. Figure 4 shows the contrast diagram of UCL intensity including the perfect complementary target (T1) with concentration of 1 fM, single-base mismatch target (1MT) and non-complementary (NC) sequence at same concentration. Test results show that the percentage of UCL intensity about 1MT is 8.2% (8.34 a.u.) and NC is only 4.5% (8.34 a.u.) compared to blank treatment (0%, 1.44 a.u.) and T1 (100%, 101.36 a.u.). And the weak UCL signals of a, b and c samples may be nonspecific adsorption resulting from incomplete ExoIII reaction or the DNA molecule on the UCNPs surface. So, the proposed biosensor shows high specificity and high selectivity for target T1. In addition, the stability of the biosensor was further investigated. The UCL intensity of T1 was detected again after the modified pore plate was immersed in buffer solution at 4 °C for 24 h. The results show that the UCL intensity of T1 has no significant differences after it had been processed. Therefore, the high stability can make the proposed biosensor accurately discriminate the complementary sequence from NC sequences, which is mostly beneficial for the ultra low detection of T1 in clinical sample.

### Analysis of serum samples

Under the optimal experimental conditions, the simulation test of c-erbB-2 oncogene in synthetic serum sample was carried out. As shown in Table 1, the recovery (Rec.) is 96 ~ 112%, and the relative standard deviation (RSD.) is 2.6 ~ 4.2% after five repetitive measurements, which indicate that the reproducibility of the assay is feasible.

**Figure 5:**
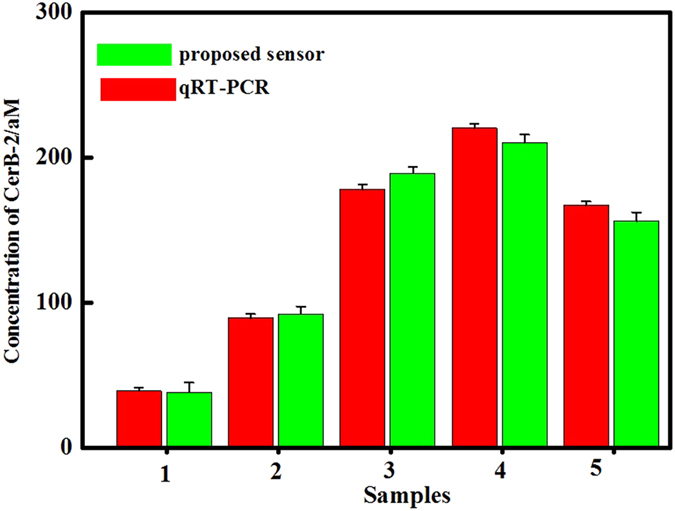
Bars represent the concentration of c-erbB-2 oncogene in serum samples detected by UCL biosensor (green bars) and qRT-PCR (red bars), respectively. Error bars represent standard deviations for measurements taken from at least five independent experiments. Tumor staging of 5 clinical samples are pT1 (sample 1 and 2), pT2 (sample 3 and 5), pT3 (sample 4) respectively.

Furthermore, the designed UCL biosensor was applied for the detection of c-erbB-2 oncogene in serum samples by using standard addition method. The serum sample 1 of breast cancer patient was chosen as an example. A series of synthetic c-erbB-2 oncogenes were spiked into serum sample 1 in equal volumes to establish a calibration curve. The concentration of c-erbB-2 oncogene in the original serum sample 1 was calculated to be 38 aM (as shown in Supplementary Fig. S2). And by the same way, the concentrations of c-erbB-2 oncogene in the other four serum samples were also obtained to be 92, 189, 210 and 156.3 aM, respectively. As shown in Fig. 5, the qRT-PCR analysis of c-erbB-2 oncogene for the same samples identified that the experimental errors of two methods located in the same range. Therefore, the proposed biosensor can be effectively used for ultra low detection of c-erbB-2 oncogene in clinical sample, which would provide credible basis for the early diagnosis and treatment of breast cancer.

## Conclusion

In summary, we have designed a new UCL biosensor based on dual signal amplification of ExoIII-assisted target cycles and long-range self-assembled DNA concatamers combined with UCNPs. The proposed biosensor has the superior stability, good sensitivity and specificity during the detection of c-erbB-2 oncogene. The LOD based on 3ơ method can reach 40 aM and RSD is 2.6 ~ 4.2%. The detection results of clinic samples demonstrate that the UCL biosensor has good reproducibility and high accuracy of the assay. Therefore, the proposed biosensor would be expected to be used for ultrasensitive detection of c-erbB-2 oncogene in clinical sample and satisfy the need of early clinical diagnosis of breast cancer in the future.

## Materials and Methods

### Materials

All chemicals were used as received without further purification. Yttrium oxide (99.999%), ytterbium oxide (99.999%) and erbium oxide (99.999%) were provided from Institute of Applied Chemistry Chinese Academy Sciences (Changchun, China). NaF and stearic acid were obtained from Sinopharm Chemical Reagent Co., Ltd (Shanghai, China). Oleic acid (OA) and 1-octadecene (ODE) were purchased from Aladdin Reagent Co., Ltd (Shanghai, China). Exonuclease III (ExoIII), bovine serum albumin (BSA) and tris(hydroxymethyl)aminomethane were purchased from New England Biolabs Co., Ltd (Beijing, China). All aqueous solutions were prepared in ultrapure water (purified by Milli-Q biocel from Milli-pore China Ltd.). DNA sequences were purchased from Sangon Biotechnology Co., Ltd (Shanghai, China) and were illustrated in Table 2. The serum samples were obtained from all participants via an institutional consent form. The study and this consent procedure were approved by the ethics committee of Fujian Medical University Union Hospital (Ethical certification No.E2014021). We confirm that all experiments were performed in accordance with relevant guidelines and regulations.

### Characterization

The size and morphology of as-prepared UCNPs were observed by a JEM-1200EX transmission electron microscope (TEM, JEOL Ltd., Japan) equipped with an electron diffractometer (ED). The crystal phase of UCNPs was identified by a Mini Flex II X’ Pert Pro diffractometer (Riguka Co., Japan) with graphite monochromatized Cu Kα radiation (λ = 0.15406 nm). The UV–vis absorption measurements were conducted on a UV2450 UV–vis spectrophotometer (Shimadzu Scientific Instruments Inc., Japan). The Zeta potentials were measured by a PSS Z3000 laser nanoparticle/electric potential meter (PSS, USA). The UCL were recorded with a Cary Eclipse fluorescence spectrophotometer (Varian Co., USA) attached an external 980 nm laser (CNI Co., China) instead of internal excitation source.

### Synthesis of NaYF_4_:Yb^3+^/Er^3+^ UCNPs

Firstly, the rare earth stearates (C_17_H_35_COO)_3_RE (RE = Y_0.78_Yb_0.20_Er_0.02_) were synthesized by reacting RE oxides with stearic acid according to the related literature^37^. Then NaYF_4_:Yb^3+^/Er^3+^ nanocrystals were synthesized by thermal decomposition^38^ of rare-earth stearates as precursors in OA-ODE system with high boiling points. In a typical procedure, 1.0 mmol of rare earth stearates were dissolved in a 100 ml three-necked flask with existence of OA (12 mL) and ODE (8 mL). After adding 24 mmol NaF, the mixture was then heated to 140 °C with vigorous stirring under vacuum for 60 minutes to form a clear solution. Afterwards, the solution was heated to 315 °C quickly and maintained at 315 °C for 45 minutes to obtain the UCNPs. The UCNPs were deposited by adding cyclohexane/ethanol (1:1 v/v) solution and separated via centrifugation. The resulted UCNPs were further purified by excess ethanol, then dried under vacuum for 24 h, finally dispersed in proper amount of chloroform.

### DNA functionalization of NaYF_4_:Yb^3+^/Er^3+^ UCNPs

The 25 nm β-NaYF_4_:Yb^3+^/Er^3+^ UCNPs were modified by applying DNA to replace OA directly according to the reference^31^. Firstly, the DNA sequence (AP2) was pre-processed as the following steps. AP2 was centrifuged for 15 min at 4000 r/min in a tabletop microcentrifuge and diluted to the required concentration. Then the solution was vortexed for several minutes and stored at 4 °C. The 20 μmol OA-coated UCNPs in 1.0 mL of chloroform was slowly added into 2 mL water solution (containing 200 nmol AP2), and the mixture was vigorously stirred overnight. Afterward, the UCNPs could be clearly transferred into the upper water layer from the chloroform layer by the attachment of DNA. After vigorously sonication, excess DNA in water solution was removed from AP2-UCNPs by centrifugation and washing. The resulted AP2-UCNPs were re-dispersed in the buffer and stored at 4 °C for further use.

### Coating of avidin in microplate and fixation of CP probes

Every pore of the 96-well plates were coated with 100 μL avidin, which was diluted by carbonate buffer (pH, 9.0). The coating reaction was sustained in refrigerator, at 4 °C for 10 h. Then the microplates were rinsed several times by phosphate buffer saline (PBS) buffer (pH, 7.4) and then dried. Finally, the microplates were sealed up with 3% BSA. Before the fixation of CP, CP were heated to 95 °C for 10 min and naturally cooled to room temperature. Then 10 uM CP were transferred to the microplates and sustained at 25 °C for 30 min. Finally, the residuals were poured out and the microplate was rinsed by PBS and baked.

### The UCL test of c-erbB-2 oncogene

The 100 uL c-erbB-2 oncogene (T1) was transferred to the 96-well plates fixed with CP. The above microplates were incubated at room temperature for 120 min after adding hybrid buffer. After the residuals were poured out, and the microplates were rinsed several times by PBS and dried. Then the proper amount of ExoIII were added in the microplates and sustained at 37 °C for 30 min. Finally, 1 μM AP1 and 1 μM AP2-UCNPs were transferred to the microplates and sustained at 25 °C for 120 min. Then the residuals were poured out, and the microplates were rinsed by PBS and baked. The UCL was detected by Cary-50 fluorescence spectrophotometer with external 980 nm laser after adding 100 L buffer into every microplate.

## Additional Information

**How to cite this article**: Lan, J. *et al.* A upconversion luminescene biosensor based on dual-signal amplification for the detection of short DNA species of c-erbB-2 oncogene. *Sci. Rep.* **6**, 24813; doi: 10.1038/srep24813 (2016).

## Supplementary Material

Supplementary Information

## Figures and Tables

**Table 1:** Determination of T1 in artificial serum samples (n = 5).

T1 added (fM)	Detected Results (fM)	Rec. (%)	RSD. (%)
0.5	0.48	96	2.6
5	5.6	112	3.8
50	52	104	4.2

**Table 2:** DNA sequences in experiments.

capture probe (CP):	5′-biotin-AAA AAT TTA TTT GAT AGG CGA ACT ATT TGT TTT AAT ATC AAA TAA TGG TT-3′
auxiliary probe 1 (AP1):	5′-CAA AAT ATA T GA TAG GCG AA-3′
auxiliary probe 2 (AP2):	5′-ATA TAT TTT GTT CGC CTA TC-3′
c-erbB-2 oncogene (T1):	5′-AAC CAT TAT TTG ATA TTA AAA CAA ATA GGC TTG-3′
single-base mismatch target (1MT):	5′-AAC CAT TAT TTG ATA TAA AAA CAA ATA GGC TTG-3′
two-base mismatch target (2MT):	5′-AAC CAT TAT TTG ATA TAA AAA CAA ATA CGC TTG-3′
noncomplementary sequence (NC):	5′-TTA GTA ATC CCC TAC AGT TTT GTC GAT CCG AAC-3′

Note: The above underlined sections are the mismatch sites.
