# Supplementary material for: A upconversion luminescene biosensor based on dual-signal amplification for the detection of short DNA species of c-erbB-2 oncogene
Source: Sci Rep. 2016 Apr 21;6:24813. doi: 10.1038/srep24813 (PMC4838860; doi:10.1038/srep24813)
Supplement: Supplementary Information [file srep24813-s1.pdf]

Electronic Supplementary Information

A upconversion luminescene biosensor based on dual-signal amplification for the detection of short DNA species of c-erbB-2 oncogene

Jianming Lan[1], Yingxin Liu[1], Li Li[1], Fadi Wen[1], Fang Wu[1], Zhizhong Han[1], Weiming Sun[1], Chunyan Li[1]* & Jinghua Chen[2]*

[1]Department of Basic Chemistry, the School of Pharmacy, Fujian Medical University, Fuzhou, Fujian 350108, P. R. China.

[2]Department of Pharmaceutical Analysis, the School of Pharmacy, Fujian Medical University, Fuzhou, Fujian 350108, P. R. China.

1. The supplementary figures

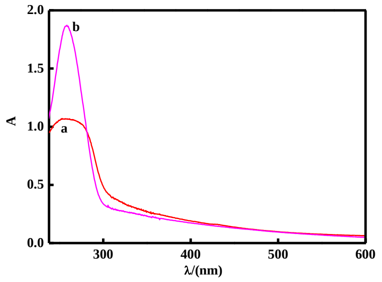

Fig. S1. The UV/Vis spectra of different solutions: (a) AP2-UCNPs, (b) DNA.

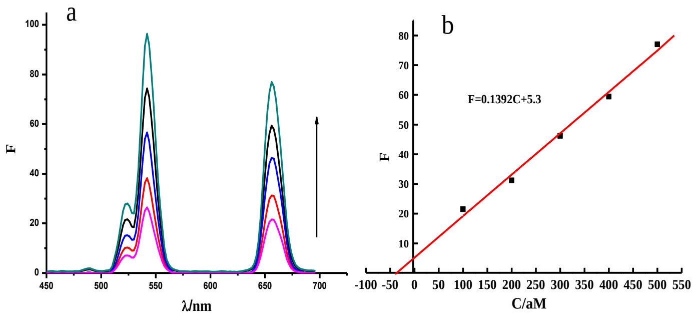

Fig. S2. (a) Variation diagram of UCL intensity with the different concentrations of c-erbB-2 oncogene of sample 1. From bottom to top: 100 aM, 200 aM, 300 aM, 400 aM, 500 aM; (b) The linear graph for UCL intensity with the concentrations of c-erbB-2 oncogene.

2. The optimization of experimental conditions

During the experiment, buffer system and pH value, temperature and time of hybridization reaction, the concentrations of CP, AP1, AP2 and ExoIII were optimized respectively. Finally we obtained the optimal experimental conditions as following. The hybridization reaction temperature was 37 ℃ and hybridization time was 2 h, the concentration of CP was 2 uM, the concentration of ExoIII is 10 U, the degradation reaction time of ExoIII was 0.5 h, the concentrations of the AP1 and AP2 were 1 uM, the time of long-range self-assembly was 2 h, and the Tris-HCl (pH 7.0) was chosen as buffer system.

3. The supplementary table

Table S1 The comparison table of LOD according to different detection methods

Order	Method	LOD	Source
1	Based on FRET from pyrene excimer to DNA intercalator SYBR green I.	10 nM	Reference[32]
2	The label-free colorimetric DNA detection strategy based on nicking endonuclease (NEase)-assisted activation of HRP-DNAzymes (NEAADNAzymes).	10 pM	Reference[33]
3	The electrochemical method based on nicking endonuclease assisted electrochemistry signal amplification (NEAESA).	0.167 pM	Reference[34]
4	The multiplex electrochemical detection of DNA based on enzyme functionalized Au nanoparticles (AuNPs).	0.1 pM	Reference[35]
5	Based on graphene quantum dots utilizing cascade amplification by nicking endonuclease and catalytic G-quadruplex DNAzyme.	30 fM	Reference[36]
6	The UCL biosensor based on dual-signal amplification of ExoIII-assisted target cycles and long-range self-assembly DNA concatamers.	40 aM	Our paper
